# Melatonin Application Improves Salt Tolerance of Alfalfa (*Medicago sativa* L.) by Enhancing Antioxidant Capacity

**DOI:** 10.3390/plants9020220

**Published:** 2020-02-08

**Authors:** Huifang Cen, Tingting Wang, Huayue Liu, Danyang Tian, Yunwei Zhang

**Affiliations:** College of Grassland Science and Technology, China Agricultural University, Beijing 100193, China; cenhuifangqk@163.com (H.C.); 17835423011@163.com (T.W.); liuhuayue@cau.edu.cn (H.L.); tdy_1993@163.com (D.T.)

**Keywords:** alfalfa, melatonin, salt tolerance, antioxidants

## Abstract

Alfalfa (*Medicago sativa* L.) is an important and widely cultivated forage grass. The productivity and forage quality of alfalfa are severely affected by salt stress. Melatonin is a bioactive molecule with versatile physiological functions and plays important roles in response to various biotic and abiotic stresses. Melatonin has been proven efficient in improving alfalfa drought and waterlogging tolerance in recent studies. In our reports, we applied melatonin exogenously to explore the effects of melatonin on alfalfa growth and salt resistance. The results demonstrated that melatonin application promoted alfalfa seed germination and seedling growth, and reduced oxidative damage under salt stress. Further application research found that melatonin alleviated salt injury in alfalfa plants under salt stress. The electrolyte leakage, malondialdehyde (MDA) content and H_2_O_2_ content were significantly reduced, and the activities of catalase (CAT), peroxidase (POD), and Cu/Zn superoxide dismutase (Cu/Zn-SOD) were increased with melatonin pretreatment compared to control plants under salt stress with the upregulation of genes related to melatonin and antioxidant enzymes biosynthesis. Melatonin was also involved in reducing Na^+^ accumulation in alfalfa plants. Our study indicates that melatonin plays a primary role as an antioxidant in scavenging H_2_O_2_ and enhancing activities of antioxidant enzymes to improve the salt tolerance of alfalfa plants.

## 1. Introduction

Alfalfa (*Medicago sativa* L.) is an excellent perennial leguminous grass and one of the most important forage crops which is widely cultivated around the world. For its high protein content, rich nutrition value and high biomass yield, it is known as the “King of Forages”. Approximately 32.2 million hectares of alfalfa is planted worldwide, and with the development of livestock industries and new policies in China, alfalfa has become the most important and widely used forage grass in integrated farming systems in China [1]. The planting area of alfalfa is about 3.77 million hectares in China, the highest among all other artificial grasslands [2,3]. 

With the autotrophic sessile nature of plants, they are continually challenged by various biotic and abiotic stresses during their growth and development stages. To defend themselves against unfavorable environmental conditions, plants have developed various complex regulation strategies, including enzymatic and non-enzymatic systems [4,5]. The enzymatic system consists of a series of antioxidant enzymes, such as catalase (CAT), peroxidase (POD), superoxide dismutase (SOD), ascorbate peroxidase (APX), and glutathione reductase (GR), which play major roles in reactive oxygen species (ROS) scavenging [6]. However, there are approximately 800 million hectares of the world’s irrigated land affected by saline or sodic globally [7,8]. Salinity has become one of the most important environmental stress factors impairing worldwide agricultural productivity. Under salinity conditions, the seed germination, growth and development processes of alfalfa are inhibited and they finally impair the herbage yield, as well as the forage quality [9]. Breeding new alfalfa cultivars with high salt tolerance is always needed. Genetic engineering and conventional breeding have been proven efficient for improving salt tolerance of various plant species [10,11,12,13]. However, they are time-consuming and complicated. Exogenous application of certain plant growth regulators such as phytohormone and other small molecules has been proven efficient at overcoming the harmful effects of salt stress on plants [14,15,16,17]. Moreover, plant growth regulators in low concentrations always play a role in plants, and are cost-effective. Foliar spraying of salicylic acid (SA) on faba bean inhibits Na^+^ accumulation and lipid peroxidation, improving the antioxidant resistance and finally mitigating the damage caused by salinity [18]. Exogenously applied poly-γ-glutamic acid on wheat maintains the Na^+^ and K^+^ homeostasis and enhances antioxidant capacity by alleviating salinity damage under salt stress conditions [19]. Exogenous spermidine application to salt-stressed cucumber improves the photosynthetic capacity and the activity of key enzymes for CO_2_ fixation by regulating the expression of related genes, and tolerance to salinity is thus conferred [20].

Melatonin (N-acetyl-5-methoxytryptamine) is a bioactive molecule that was first identified in the mammalian pineal gland. It is a derivate from the essential amino acid tryptophan. Melatonin plays an important role in regulating circadian rhythms, sleep disorders, immunologic enhancement and antioxidative activity in animals [21,22]. Melatonin was first identified in plants in 1995, and since then there has been extensive research seeking to reveal the presence of melatonin in the plant kingdom and the physiological roles melatonin plays in plants [23]. Melatonin has been detected in a considerable variety of plants consisting of vegetables, cereals, fruits and medicinal herbs [24,25]. Melatonin levels vary from a few pg to over several hundred µg per fresh weight between species, organs, and different environment conditions [25]. Melatonin has proven to be a versatile regulator and participates in plant growth, development and stress responses. It participates in seed germination, lateral root formation, flowering, circadian rhythms, photosynthesis, senescence and response to various environmental stresses [26,27,28,29,30]. The biosynthesis of melatonin in plants is catalyzed by four successive enzymatic steps, including tryptophan decarboxylase (TDC), tryptamine 5-hydroxylase (T5H), serotonin N-acetyltransferase (SNAT), and N-acetylserotonin methyltransferase (ASMT)/caffeic acid O-methyltransferase (COMT) [31]. The upregulation of these genes in melatonin biosynthesis pathway is beneficial for endogenous melatonin accumulation.

Evidence shows that melatonin is a powerful antioxidant in plant responses to abiotic stresses [32,33]. Under stress conditions, plants produce reactive oxygen species (ROSs) and excessive ROSs inevitably lead to oxidative damage [34]. Experimental evidence proves that melatonin is an efficient free-radical scavenger and antioxidant under stress conditions, can directly scavenge excessive ROS and reactive nitrogen species (RNS), and can enhance the activity of the antioxidative enzymatic system, which controls the burst of hydrogen peroxide in plants and protects them from oxidative stress [35,36]. Melatonin has been used extensively in enhancing multiple stress resistances in various plant species including rice, wheat, barley, cucumber, soybean, perennial ryegrass and alfalfa [37,38,39,40,41,42,43]. Melatonin participates in stress responses via crosstalk with various phytohormones, such as auxin (IAA), ethylene (ET), jasmonic acid (JA), salicylic acid (SA) and brassinosteroids (BR) [33]. The crosstalk of melatonin with other phytohormones occurs through regulating the expression of its upstream and downstream genes. Melatonin modulates the salt tolerance of grapevines by enhancing ethylene biosynthesis via regulating the transcripts of ACS1 [44]. Melatonin regulates the salt tolerance of sunflower by accompanying with NO to modulate the expression of *Cu/Zn-SOD* and *Mn-SOD* genes [45]. Exogenous applications of melatonin alleviate oxidative damage induced by salt stress by enhancing the expression of genes related to ABA and GA biosynthesis [46]. Pretreatment with melatonin alleviates the growth inhibition and oxidative damage of *M. hupehensis* by directly scavenging H_2_O_2_ and enhancing the activities of antioxidative enzymes, and by controlling the expression of ion-channel genes to maintain homeostasis [47]. Melatonin has been reported to improve the drought tolerance and waterlogging resistance by exogenous application on alfalfa [43,48]. Based on the existing reports, we assumed that the exogenous application of melatonin might be effective in improving the salt tolerance of alfalfa.

To validate the hypothesis, we assessed the effects of various concentrations of melatonin on the germination and seedling growth of alfalfa under salinity conditions. The results showed that the exogenous application of melatonin can remarkably enhance salt tolerance and alleviate the salt injury of alfalfa. Our study confirms that melatonin performs its primary function as an antioxidant, positively improving the salt tolerance of alfalfa through scavenging reactive oxygen and enhancing the activities of antioxidant enzymes.

## 2. Results

### 2.1. Melatonin Promotes Seed Germination and Seedling Growth under Salt Stress

**Figure 1:**
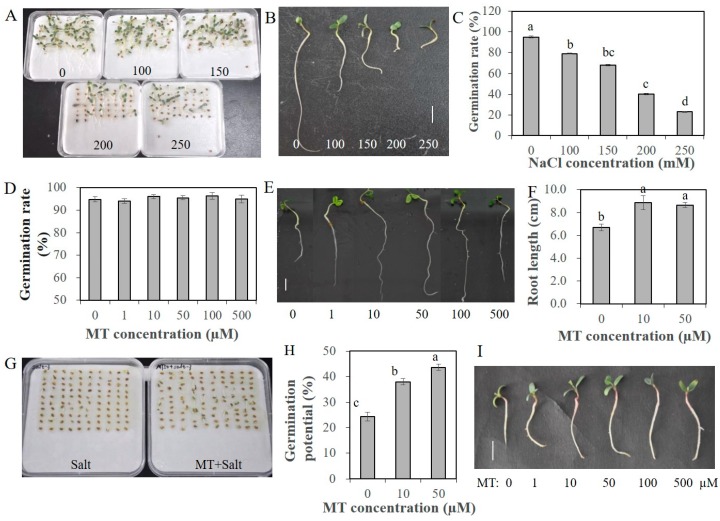
Germination status of alfalfa seeds under various concentrations of salt and melatonin (MT). (**A**), Alfalfa seeds germinated under salt stress with various concentrations of NaCl (0, 100, 150, 200 and 250 mM) for 7 days; (**B**), Seedlings after being germinated under different concentrations of NaCl (0, 100, 150, 200 and 250 mM) for 7 days; (**C**), Germination rates of alfalfa seeds germinated under various concentrations of NaCl (0, 100, 150, 200 and 250 mM) for 7 days; (**D**), Germination rate of alfalfa seeds pretreated with various concentrations of MT (0, 1, 10, 50, 100 and 500 µM) and germinated under normal conditions for 7 days; (**E**), Seedlings germinated under normal conditions for 7 days with different concentrations of MT (0, 100, 150, 200 and 250 mM) pretreatment; (**F**), Root length of alfalfa seedlings after being germinated under normal conditions for 7 days with several concentrations of MT (0, 10 and 50 µM) pretreated; (**G**), Alfalfa seeds germinated under salt stress (200 mM NaCl) and salt stress with MT pretreatment (50 µM MT + 200 mM NaCl) for 4 days; (**H**), Germination potential of alfalfa seeds with MT (0, 10 and 50 µM) pretreated and germinated under salt stress with 200 mM NaCl for 4 days; (**I**), Seedlings germinated under 200 mM NaCl condition for 7 days with MT (0, 1, 10, 50, 100 and 500 µM) pretreatment, scale bar, 1 cm. Data are represented as means ± SE (*n* = 3), and bars with different letters indicate the differences between these different treatment groups according to ANOVA analysis (*p* < 0.05).

**Figure 2:**
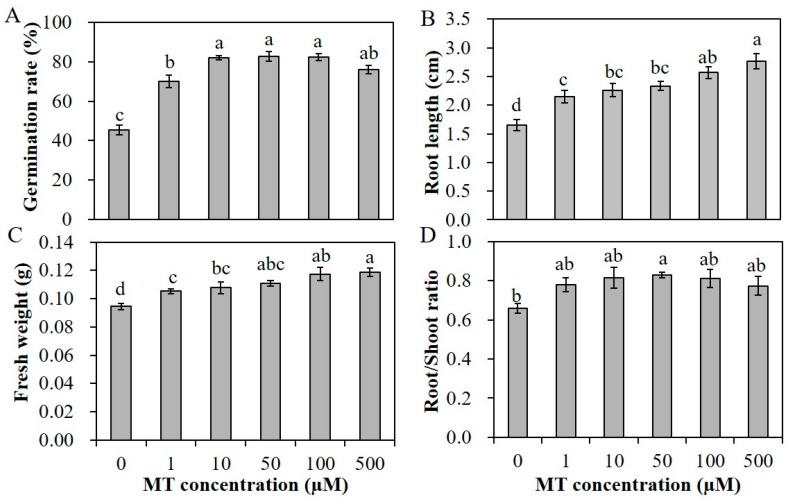
Germination rate (**A**), root length (**B**), fresh weight (**C**) and root/shoot ratio (**D**) of alfalfa seedlings pretreated with various concentrations of MT (0, 1, 10, 50, 100 and 500 µM) and germinated under 200 mM NaCl for 7 days. Data are represented as means ± SE (*n* = 3), and bars with different letters indicate the differences between these different treatment groups according to ANOVA analysis (*p* < 0.05).

Melatonin was proved beneficial to plant growth and development, as well as response to various abiotic stresses. Before salinity treatment, an appropriate salt and melatonin (MT) concentration was screened. The germination rate of alfalfa seeds under 200 mM and 250 mM NaCl was about 40% and 23%, respectively (Figure 1A,C). The root length of seedlings under salinity conditions was shorter than that under normal conditions (Figure 1B). Therefore, we chose 200 mM NaCl in the following salinity treatment. To assess the effectivity of MT on seed germination and seedling growth, alfalfa seeds pretreated with different concentrations of MT (0, 1, 10, 50, 100 and 500 µM) were germinated under non-stressed conditions. MT promoted the elongation of seedling roots significantly; the root length of seedlings with 10 and 50 µM MT pretreatments was at least 9 cm, remarkably longer than 6.5 cm of the control seedlings (Figure 1E,F). However, the germination rate of alfalfa seeds under normal conditions was almost 95%; pretreatment with MT could not further promote the germination rate (Figure 1D). Under salinity condition, primed seeds showed a superior germination potential, and the seed coats broke earlier. The increases in germination potential with 10 and 50 µM MT pretreatment vary from 13.2% to 19.9%, respectively, after 4 days for seed germination (Figure 1G,H). After 10 days, the germination rate of seeds with 1 µM MT treatment was 69.8%, rates with 10–100 µM MT treatments were up to 82%, and the rate with 500 µM MT decreased to 75.6% (Figure 2A). In addition, the seedling roots were also elongated compared to unprimed seedlings (Figure 1I). The root length of primed seedlings under 200 mM NaCl varied from 2.10 to 2.70 cm with various concentration of MT pretreatment, on average 1 cm longer than unprimed seedlings (Figure 2B). The fresh weights of seedlings with various concentrations of MT pretreatment were all higher than those of unprimed seedlings, and the root/shoot ratio was also increased slightly (Figure 2C,D). The results indicated that melatonin can alleviate the seedling growth inhibition caused by salt stress. 

### 2.2. Melatonin Reduces Salt Injury of Alfalfa Seedlings under Salt Stress

**Figure 3:**
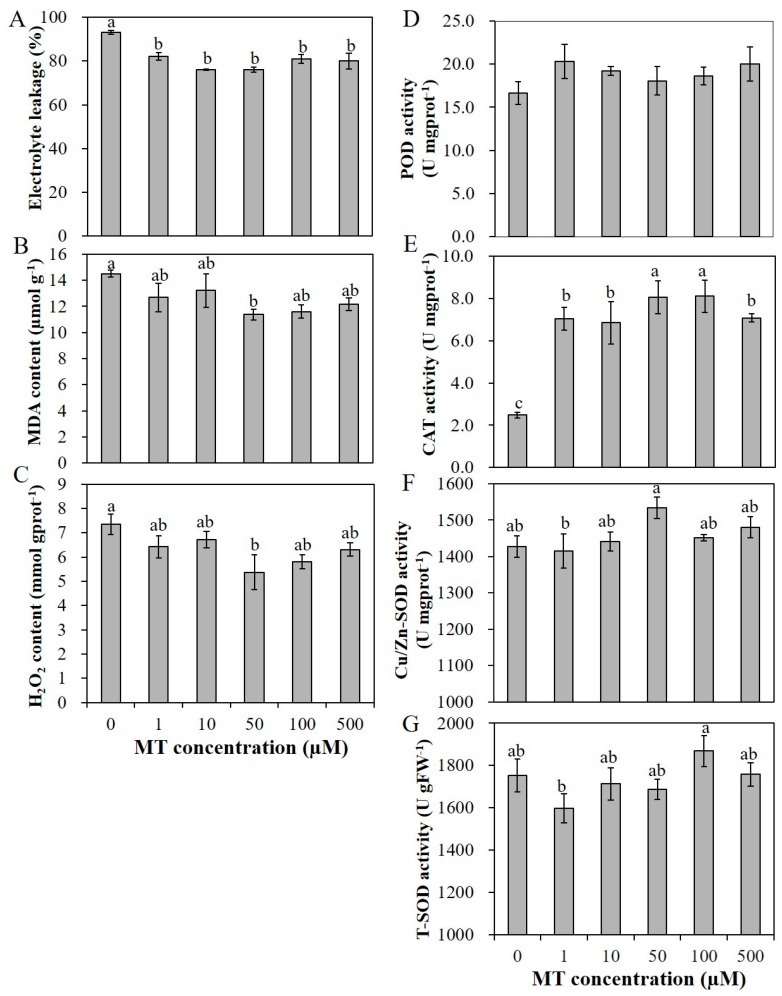
The mitigated effects of various concentrations of MT (0, 1, 10, 50, 100 and 500 µM) on electrolyte leakage (**A**), malondialdehyde (MDA) content (**B**), H_2_O_2_ content (**C**) and the enzyme activities of peroxidase (POD) (**D**), catalase (CAT) (**E**), Cu/Zn superoxide dismutase (Cu/Zn-SOD) (**F**), and T-SOD (**G**) of alfalfa seedlings under 200 mM NaCl salinity condition. Data are represented as means ± SE (*n* = 3), and bars with different letters indicate the differences between these different treatment groups according to ANOVA analysis (*p* < 0.05).

The application of melatonin promoted the seedling growth under salt stress with 200 mM NaCl. Salt stress affects the stability of plant cell membranes. Electrolyte leakage (EL) represents the plant cell permeability, which is an important index of a plant’s salt tolerance [49]. Malondialdehyde (MDA) is the end product of lipid oxidation, and reflects the degree of membrane lipid peroxidation. These indicators can indirectly present the damage degree of the membrane system and the stress resistance of plants [49]. Compared with the unprimed seedlings, primed seedlings integrally exhibited significantly lower EL, lower MDA content and lower H_2_O_2_ content especially with 50 µM MT pretreatment (Figure 3A–C). Despite that, the activities of antioxidant enzymes CAT and POD were significantly increased and the activity of Cu/Zn-SOD with 50 µM MT pretreatment was also significantly higher than the unprimed plants, while the activity of total superoxide dismutase (T-SOD) exhibited no significant difference between MT pretreatment and control seedlings (Figure 3D–G). To summarize, pretreatment with MT enhanced the salt tolerance of alfalfa seedlings by reducing the cell permeability and membrane lipid peroxidation, and by reducing the H_2_O_2_ accumulation, and 50 µM MT exhibited the optimal effects, so it was chosen for further applications.

### 2.3. Melatonin Application Improves Salt Tolerance of Alfalfa Plants

**Figure 4:**
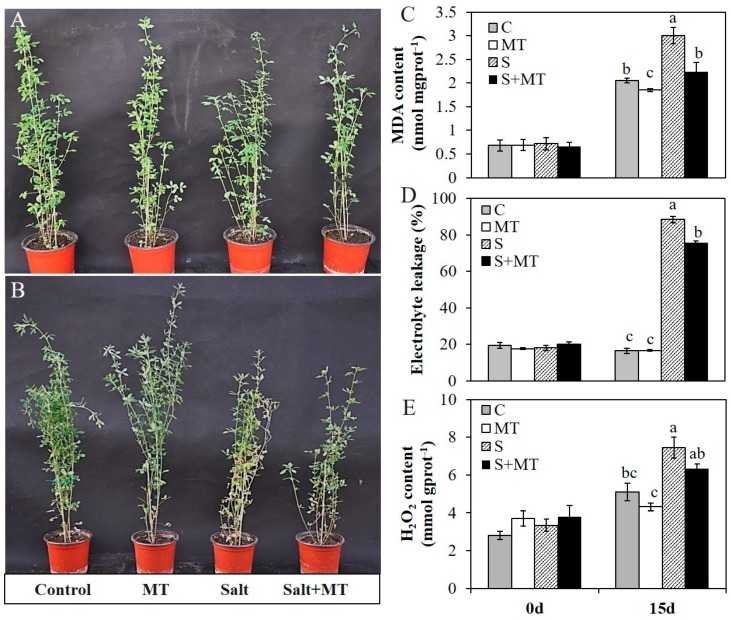
Effects of MT (50 µM) pretreatment on one-month-old alfalfa plants exposed to salt stress (200 mM NaCl) for 15 days. A, B, Plants sprayed with MT (50 µM) or water for 7 days (**A**) and subsequently subjected to salt stress with 200 mM NaCl or normal conditions for 15 days (**B**). Plants from left to right: plants grown under normal conditions, plants pretreated with MT and grown under normal conditions, plants subjected to salt stress, and plants pretreated with MT and then subjected to salt stress; C–E, Effects of MT pretreatment on the MDA content (**C**), electrolyte leakage (**D**) and H_2_O_2_ content (**E**) of alfalfa plants before and after salinity treatment or under normal conditions. Data are represented as means ± SE (*n* = 3), and bars with different letters indicate the differences between these four different groups according to ANOVA analysis (*p* < 0.05).

To assess the effects of melatonin on alfalfa plants, we applied 50 µM MT to one-month-old plants via foliar spraying for seven days. Half of the MT-pretreated plants and half of the control plants were subjected to salinity treatment with 200 mM NaCl. Fifteen days later, melatonin-treated plants under normal conditions were robust, which was similar to the control plants. Plants under salt stress showed retarded growth and with visible foliar injury, and a majority of leaves were wilted. However, melatonin-treated plants exposed to salt stress for 15 days exhibited obvious mitigating effects (Figure 4A,B).

**Figure 5:**
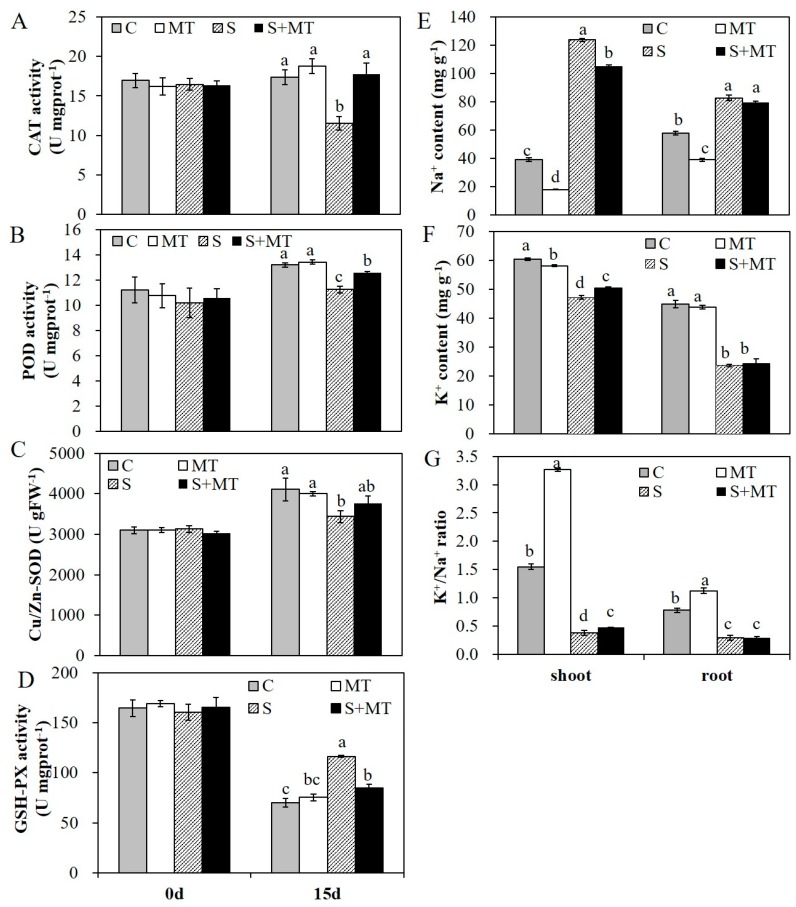
The activities of antioxidative enzymes and the Na^+^, K^+^ contents of alfalfa plants under normal or salt stress (200 mM NaCl) conditions. A–D, the activities of CAT (**A**), POD (**B**), Cu/Zn-SOD (**C**) and glutathione peroxidase (GSH-PX) (**D**) in different groups of plants before and after salt stress treatment; E–G, Na^+^ content (**E**), K^+^ content (**F**), and the K^+^/Na^+^ ratio (**G**) in the shoots and roots of one-month-old alfalfa plants pretreated with MT and exposed to salt stress with 200 mM NaCl for 15 days. Data are represented as means ± SE (*n* = 3), and bars with different letters indicate the differences between these four different groups according to ANOVA analysis (*p* < 0.05).

Before salinity treatment, MT pretreatment had no effect on these physiological indexes and they all remain at a relative low level without significant differences. Fifteen days later, MDA content in all these groups were increased, while MDA content in plants pretreated with MT were significantly lower than that in control plants either under normal conditions or salinity conditions (Figure 4C). With 200 mM of NaCl treatment for 15 days, the electrolyte leakage of alfalfa plants was significantly increased; the electrolyte leakage had reached 88.4% with salt stress treatment, and reached 75.3% in plants with MT pretreatment (Figure 4D). The H_2_O_2_ content was also increased in all four group plants after 15 days, while that in all plants with MT pretreatment was significantly lower than that in control plants under normal or salinity conditions (Figure 4E). In addition, the activities of several antioxidant enzymes were also improved with MT pretreatment. Before salinity treatment, there were no significant differences in any of these plants, which demonstrated that MT pretreatment had no significant effects on the activities of these antioxidant enzymes under normal conditions. Under salt stress treatment with 200 mM NaCl for 15 days, the activities of CAT, POD and Cu/Zn-SOD in plants with MT pretreatment were significantly higher than that in salt-treated plants (Figure 5A–C). However, the activity of glutathione peroxidase (GSH-PX) was decreased in all plants after salinity treatment, and the activity of GSH-PX in plants with MT pretreatment was significantly lower than that in salt-treated plants (Figure 5D). Meanwhile, salt stress severely affected ion homeostasis, and plants could accumulate excessive Na^+^ and lose K^+^ in cells, causing ion toxicity. To detect the effects of MT on ion homeostasis, we measured Na^+^ and K^+^ content in shoot and root parts in these plants under normal or salt stress conditions for 15 days. The results showed that the Na^+^ content in plants with salt stress treatment was significantly higher than that in non-stressed plants, and MT pretreatment reduced Na^+^ accumulation both in shoot and root parts, in both salinity and normal conditions, especially in the shoot parts of alfalfa plants (Figure 5E). Salt stress treatment reduced K^+^ uptake in both shoots and roots, and MT pretreatment improved K^+^ uptake only in shoot parts under salt stress conditions (Figure 5F). For the K^+^/Na^+^ ratio, plants with MT pretreatment under normal conditions exhibited a significantly higher K^+^/Na^+^ ratio both in shoot and root parts. Under salt stress conditions, the K^+^/Na^+^ ratio was increased in the shoot part of the plants pretreated with 50 µM MT (Figure 5G). The results showed that MT mitigated salinity damage mainly by protecting membrane stability, enhancing antioxidative enzyme activities and reducing Na^+^ accumulation in alfalfa plants.

### 2.4. Melatonin Application Induces the Expression of Genes Related to Melatonin and Antioxidants Biosynthesis

**Figure 6:**
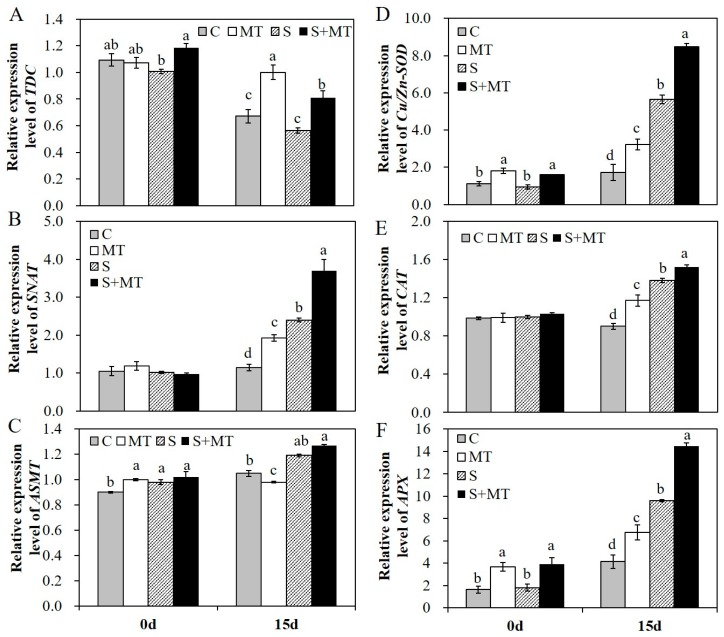
Relative expression level of several selected genes in the melatonin biosynthesis pathway and genes encoding antioxidative enzymes involved in reactive oxygen species (ROS) metabolism A–C, relative expression level of tryptophan decarboxylase (*TDC*) (**A**), serotonin N-acetyltransferase (*SNAT*) (**B**), and N-acetylserotonin methyltransferase (*ASMT*) (**C**) in the melatonin biosynthesis pathway in different treated plants before and after 200 mM NaCl treatment for 15 days; D–F, relative expression level of *Cu/Zn-SOD* (**D**), *CAT* (**E**), and ascorbate peroxidase (*APX*) (**F**) in leaves of alfalfa plants before and after 200 mM NaCl treatment for 15 days. Data are represented as means ± SE (*n* = 3), and bars with different letters indicate the differences between these four different groups according to ANOVA analysis (*p* < 0.05).

To further explore the regulation mechanism of melatonin in alfalfa plants’ responses to salt stress, we detected the relative expression level of several genes in the melatonin biosynthesis pathway and of genes related to antioxidants biosynthesis by qRT-PCR. The results showed that exogenous application of MT promoted the transcript of *TDC* under normal and salt stress conditions (Figure 6A). MT application also promoted the transcript of *SNAT* in alfalfa plants, especially under salt stress conditions (Figure 6B). The transcript of *ASMT* was improved in alfalfa under salt stress treatment for 15 days, but increases in the *ASMT* transcript level under salt stress conditions were not that obvious compared to normal conditions, nor were they remarkable in plants with MT pretreatment compared to non-stressed plants (Figure 6C). Melatonin pretreatment increased the activities of antioxidant enzymes. The transcript levels of these antioxidant enzyme biosynthesis genes were evaluated in different treatment plants. The transcript levels of *Cu/Zn-SOD*, *CAT* and *APX* genes were increased significantly by MT pretreatment under salt stress, and MT application also promoted the transcripts of *Cu/Zn-SOD* and *APX* genes under normal conditions (Figure 6D–F). The results demonstrated that melatonin plays a role as a positive antioxidant in scavenging reactive oxygen and enhancing the activities of antioxidant enzymes by upregulating the expression of genes related to melatonin and antioxidants biosynthesis, thus enhancing the salt tolerance of alfalfa plants.

## 3. Discussion

Salt stress is one of the most commonly encountered abiotic stresses for plants, and soil salinization is a severe environmental problem affecting crop fields. The current research provides insight into melatonin applications enhancing salt tolerance in various crop species [50,51]. Alfalfa is one of the most important forage grasses cultivated worldwide with a high biomass yield and a high protein content. Salt stress strongly limits the growth, quality and productivity of alfalfa [6]. Melatonin is an environmentally friendly bioactive molecule, which plays important roles in plant defense against salt stress [50]. Previous reports have confirmed that exogenous melatonin promoted alfalfa drought tolerance by modulating nitro-oxidative homeostasis and proline metabolism [43] and promoted waterlogging tolerance by regulating polyamine and ethylene metabolism [48]. We previously found that overexpression of *AANAT* and *HIOMT* from *Ovis aries* in switchgrass promoted plant growth and improved the salt tolerance of transgenic switchgrass [30]. The genetic manipulation of melatonin biosynthesis genes is an efficient way to enhance the salt tolerance of transgenic plants, and it is noteworthy in terms of exogenous applications that melatonin can be brought into practice to improve the salt tolerance of plants.

The enhancement of salt tolerance through melatonin application mainly depends on directly scavenging ROS and enhancing the activities of antioxidative enzymes and photosynthetic efficiency, as well as regulating the transcription of genes related to salt stress [34]. Melatonin plays a function with dose-dependent effects, and an optimum concentration of melatonin plays a role in resisting environmental stresses; optimum concentrations are different among different plant species [52]. Before exogenous application of melatonin, a suitable melatonin concentration must be chosen through pre-experiment evaluation. Before our research, we designed germination experiments to choose suitable concentrations of NaCl and melatonin, respectively, and these were crucial to the success of the experiments. Depending on the germination rate, seedling growth, and antioxidative abilities under salt stress, we chose 50 µM melatonin for further application research. Melatonin pretreatment has no influence on plant growth under normal conditions; under salt stress conditions, melatonin plays roles in scavenging reactive oxygen and in enhancing the activities of antioxidant enzymes, mainly CAT and POD, protecting plants from stresses. The high activities of antioxidant enzymes in plants under salt stress are necessary for plants to defend against ROS damage, and the high antioxidant activities sometimes represent high oxidative tolerance. The antioxidant enzymes in the enzymatic system have been assigned a specific role in ROS detoxification. For example, SOD converted O^−^_2_ into H_2_O_2_ in plant cells, and H_2_O_2_ was then detoxified by CAT and POD. CAT catalyzes H_2_O_2_ hydrolyzed into H_2_O and O_2_, and POD has a higher affinity to H_2_O_2_ than CAT [53,54]. In our study, the higher activities of CAT and POD in melatonin-pretreated plants corresponded with reduced H_2_O_2_ accumulation under salt stress. The relatively high Cu/Zn-SOD activity and unchanged T-SOD activity in melatonin-pretreated alfalfa seedlings might indicate that, under salt stress, alfalfa seedlings mainly accumulate H_2_O_2_, rather than O^−^_2_. Glutathione peroxidase (GSH-PX) is an important peroxidase that widely exists in plants, and that belongs to the non-enzymatic system. It catalyzes glutathione (GSH) into glutathione disulfide (GSSG), causes poisonous peroxide reduction into non-toxic hydroxyl compounds and participates in H_2_O_2_ decomposition [55,56]. The activity of GSH-PX in our results with MT pretreatment was decreased under salt stress, which might have resulted from a different regulation pathway in alfalfa plants. An explanation requires further research. The relative expression levels of *Cu/Zn-SOD*, *CAT* and *APX* genes in plants with MT pretreatment were upregulated under normal and salt stress conditions, which contributed to H_2_O_2_ scavenging to enhance the antioxidative ability of plants. However, the main function of melatonin on enzymatic or non-enzymatic system-mediated ROS scavenging in alfalfa plants still requires further study. The positive effects of melatonin on antioxidant enzymes system under salt stress conditions is also confirmed in tomato seedlings and maize seedlings [57,58]. Exogenous application of melatonin promotes plant growth and stress resistance mainly through enhancing the plant endogenous melatonin level. The relative expression level of *TDC*, *SNAT* and *ASMT* genes were all upregulated with MT pretreatment under salt stress, which was dedicated to the biosynthesis of endogenous melatonin in plants. The relative expression level of *ASMT* was not increased significantly, which might have resulted from the alternative function of caffeic acid O-methyltransferase (*COMT*) in the last step of melatonin biosynthesis [59].

The current studies were mainly dedicated to the exogenous application of melatonin so as to enhance various abiotic and biotic stresses. The effects of exogenous melatonin were discrepant with differences in treatment time, treatment organs, melatonin concentration and plant species. With the identification of genes in the melatonin biosynthesis pathway, to uncover the regulation mechanisms of melatonin in plants, the manipulation of genes expression in the melatonin biosynthesis pathway, mainly *SNAT* and *ASMT*, was an efficient way to modulate endogenous melatonin and to further reveal the melatonin regulation pathway in response to various stress conditions, especially with the discovery of the first melatonin receptor CAND2/PMTR1 [60]. Based on the positive effects of melatonin on alfalfa salt tolerance, we supposed that improving endogenous melatonin content might have profound effects on alfalfa growth and abiotic stress resistance. We isolated *SNAT* and *ASMT* genes from alfalfa and overexpressed them respectively into the alfalfa genome, and the elevated melatonin content in transgenic plants promoted transgenic plant growth. In the future, we would like to evaluate the abiotic stress resistance and explore the molecular mechanism of melatonin regulation with respect to plant resistance to abiotic stresses.

In summary, we evaluated the effects of exogenous melatonin on alfalfa germination, seedling growth, and plant resistance to salt stress. The results demonstrate that exogenous application of melatonin can promote plant growth and alleviate the salt damage of alfalfa plants under salt stress by upregulating the expression of genes related to melatonin biosynthesis and antioxidative enzyme activities. The salt tolerance of alfalfa was improved by reducing oxidative damage and enhancing the activities of antioxidant enzymes, which provides insight into how alfalfa salt tolerance can be improved with a novel bioactive molecule, melatonin.

## 4. Materials and Methods 

### 4.1. Plant Materials and Regents

All of the experiments were conducted at China Agricultural University, Beijing (39.9° N, 116.3° E). The alfalfa (*Medicago sativa* L.) cultivar used in this experiment was “Zhongmu No. 2”. All chemicals used in experiments were of analytical grade. Melatonin (N-acetyl-5-methoxytryptamine) was purchased from Sigma-Aldrich (M5250, Shanghai, China) and stored at −20 °C.

### 4.2. Germination Tests

To screen the appropriate salinity concentration, the alfalfa seeds were sterilized in a 5% sodium hypochlorite solution for 15 min, and rinsed with distilled water five times. Fifty sterile seeds were placed on 12 × 12 cm Petri dishes with three layers of filter paper moistened with 10 mL of NaCl solution of various concentrations (0, 100, 150, 200 and 250 mM), germinated at 25 °C in darkness for 2 days, and then transferred to light conditions at 25/22 °C under a light/dark photoperiod of 16/8 h. One week later, the germination rates and the lengths of the seedling roots were measured. Three biological replicates were performed.

To assess the melatonin effect upon salt stress, sterile seeds were immersed into melatonin solutions of various concentrations (0,1, 10, 50, 100 and 500 µM) for 24 h at 4 °C in the dark. After being air-dried, one hundred primed seeds were placed on 12 × 12 cm Petri dishes with three layers of filter paper moistened with 10 mL of distilled water or a 200 mM NaCl solution, germinated at 25 °C in the dark for 2 days, and then transferred to light conditions at 25/22 °C under a light/dark photoperiod of 16/8 h. Three replications of 100 seeds per plate were performed. The germination potential, germination rate, root length, fresh weight and root/shoot ratio of the plants with various concentrations of MT pretreatment were also recorded.

### 4.3. Melatonin Application and Salinity Treatment of Alfalfa Plants

Seedlings germinated on the filter paper with distilled water were transferred to plastic pots (15 cm in diameter) with a mixture of soil (vermiculite/humus = 1:1) and maintained in a growth room under a 16/8 h (light/dark) photoperiod with 200 µmol m^−2^s^−2^ light intensity at 25 ± 2 °C. Seedlings were watered with 1/2 × Hoagland solution every two days. One month later, when the height of the seedlings was about 15 cm, seedlings were divided into four groups with two control groups and two experiment groups. Control Group 2 (CK2) and the Experiment Group 2 (E2) were applied with 50 mL of a 50 µM melatonin solution by foliar spraying every night, while Control Group 1 (CK1) and Experiment Group 1 (E1) were sprayed with 50 mL of deionized water every night for one week. Hereafter, CK1 and CK2 were watered with a nutrient solution every other day, and E1 and E2 were watered with a nutrient solution with 250 mM NaCl every other day for another two weeks. Each group contained 12 pots with three plants per pot.

### 4.4. Measurement of Electrolyte Leakage and Malondialdehyde (MDA) Content

Electrolyte leakage was determined according to our previous reports [61]. The fresh seedlings or leaves (0.2 g) were washed with deionized water and then immersed into 20 mL of deionized water and agitated at room temperature for 24 h. After agitation, the conductivity was measured, the samples were autoclave-sterilized for 30 min and then agitated for another 24 h at room temperature to measure the conductivity again. The electrical conductivity was measured with a conductometer (AZ pH/mV/Cond./TDS/Temp. meter 86505).

Malondialdehyde (MDA) content was extracted and measured using the MDA Kits (Suzhou Comin Biotechnology Co., Ltd., Suzhou, China; Nanjing Jiancheng Bioengineering Institute, Nanjing, China) according to the manufacturer’s instructions.

### 4.5. Measurement of Activities of Antioxidant Enzymes

The leaves of plants before and after salt stress treatment were sampled for catalase (CAT), peroxidase (POD), and superoxide dismutase (SOD) measurement with the corresponding plant kits (Suzhou Comin Biotechnology Co., Ltd. Suzhou, China) following the manufacturer’s instructions with a spectrophotometer (Hitachi UH5300, Tokyo, Japan). 

### 4.6. Measurement of Na^+^ and K^+^ Content

The Na^+^ and K^+^ content was measured according to our previous work [62]. The shoot and root parts of alfalfa after different treatments were harvested and dried at 65 °C for 48 h. Approximately 50 mg of dry powder samples were sampled into 10 mL plastic tubes and 8 mL of deionized water was added. The mixture was held in a bath of boiling water for about 30 min. The supernatant was transferred into a 50 mL plastic tube. Deionized water was added to the mixture, and the steps were repeated 3–4 times. All of the supernatant was then filtered and diluted to 50 mL with deionized water. After that, a flame spectrophotometer (Sherwood, UK) was used to measure the Na^+^ and K^+^ content. Three biological replicates were performed.

### 4.7. Extraction of Total RNA and Quantitative Real-Time PCR Analyses

Total RNA was isolated from leaves using an RNA Extraction Kit (Huayueyang Bio, Beijing, China). The integrity of the RNA was ensured by gel electrophoresis and an absorbance measurement at 260 and 280 nm with a Nanodrop 2000. The intact RNA was reverse-transcribed with the PrimeScript RT reagent Kit with gDNA Eraser (Takara, Dalian, China) according to the manufacturer’s instructions. Synthesized cDNA was subjected to PCR with an actin gene primer from alfalfa to determinate the quality [60]. The diluted cDNAs derived from the leaves of different group plants were then used as templates and were subject to real-time quantitative PCR. The primers were designed according to the gene sequence downloaded from The Alfalfa Gene Index and the Expression Atlas Database (http://plantgrn.noble.org/AGED/index.jsp). The relative expression levels of target genes were calculated with the formula 2^−ΔΔCT^ method. All of the primers used in qRT-PCR are listed in Table 1.

### 4.8. Statistical Analysis

Each experiment was repeated three times and with at least three biological replications. All data were subjected to one-way analysis of variance (ANOVA, SPSS 18.0), and multiple comparisons of the mean value were made via Duncan’s test. 

## Figures and Tables

**Table 1:** Primers list used in qRT-PCR.

Primer Name	Primer Sequences (5′-3′)
TDC-F	CTCGCAGGATCTTGTCACGG
TDC-R	AGGCACTCCTTCTGCCTCAT
SNAT-F	GTCAGAGGGGAATGAACAAAA
SNAT-R	TTCCACGACTTTACTATCTGCG
ASMT-F	ATTTCTTCACTACCAATCCACCC
ASMT-R	CCACACTCATTGGATTGTTCTAAA
Cu/Zn-SOD-F	TCCACTGGTCCTCACTTCAATC
Cu/Zn-SOD-R	GACAGCCCTTCCGAGTATGG
CAT-F	TGAAGACCCCTCCCTACGAA
CAT-R	GAACTCAGGTGAAGGATTGCC
APX-F	AACGAAACAAAATGGCAGACC
APX-R	AATTGAGCGAGGAAACGGA
Actin-F	CAAAAGATGGCAGATGCTGAGGAT
Actin-R	CATGACACCAGTATGACGAGGTCG
